# Clinical Characteristics and Treatment Outcome of 485 Patients with Nonfunctioning Pituitary Macroadenomas

**DOI:** 10.1155/2015/756069

**Published:** 2015-02-08

**Authors:** Guadalupe Vargas, Baldomero Gonzalez, Claudia Ramirez, Aldo Ferreira, Etual Espinosa, Victoria Mendoza, Gerardo Guinto, Blas Lopez-Felix, Erick Zepeda, Moisés Mercado

**Affiliations:** ^1^Endocrinology Service, Experimental Endocrinology Unit, Hospital de Especialidades, Centro Médico Nacional Siglo XXI, Instituto Mexicano del Seguro Social, Mexico; ^2^American British Hospital Neurological Center, Mexico City, Mexico; ^3^The Department of Neurosurgery, Hospital de Especialidades, Centro Médico Nacional Siglo XXI, Instituto Mexicano del Seguro Social, Mexico

## Abstract

*Background*. Nonfunctioning pituitary adenomas (NFPAs) are the most common benign lesions of the pituitary gland. *Objective*. To describe our experience with the management of NFPA. *Study Design and Methods*. Retrospective evaluation of NFPA patients managed between 2008 and 2013. We analyzed data regarding clinical presentation, imaging diagnosis, hormonal status, surgical, radiotherapeutic, and pharmacological treatment, and outcome. *Results*. 485 patients (54% men, mean age 53 ± 14 years) were followed for a median of 6.5 years. Visual field abnormalities and headaches were the presenting complaints in 87% and 66%, respectively. The diagnosis of NFPA was made incidentally in 6.2%, and 8% presented with clinical evidence of apoplexy. All patients harbored macroadenomas, with a median volume of 10306 mm^3^; 57.9% had supra- or parasellar invasion and 19.6% had tumors larger than 4 cm. Central hypothyroidism, hypogonadism, and hypocortisolism were present in 47.2%, 35.9%, and 27.4%, respectively. Surgical resection was performed at least once in 85.7%. Tumor persistence was documented in 27% and was related to the size and invasiveness of the lesion. In selected cases, radiotherapy proved to be effective in controlling or preventing tumor growth. *Conclusions*. The diagnosis and treatment of NFPA are complex and require a multidisciplinary approach.

## 1. Introduction

Clinically nonfunctioning pituitary adenomas (NFPAs) constitute over one-third of all pituitary adenomas [1–3]. They are so-called because they do not produce any hormonal hypersecretion syndrome but rather present with symptoms and signs related to the mass effect of the pituitary lesion such as headaches and visual disturbances [1–4]. NFPAs are frequently found incidentally during imaging studies performed for unrelated reasons and over a third of them show biochemical evidence of one or more anterior pituitary hormone deficiencies [1, 2]. Immunohistochemical evaluation has revealed that most of these lesions are of gonadotroph differentiation, as they usually immunostain for the beta subunit of LH and/or FSH, as well as for the common alpha subunit of these glycoproteic hormones; a small proportion of these patients have tumors that immunostain for ACTH, GH, and TSH, the so-called “silent corticotroph, somatotroph, and thyrotroph adenomas,” respectively [5, 6]. Finally, a nonnegligible proportion of these tumors do not immunostain for any peptide hormone at all and are thus called null-cell adenomas [5, 6].

The treatment of choice for NFPA is transsphenoidal surgery; although not infrequently, the tumor cannot be removed completely [1, 2, 7, 8]. The recurrence and persistence rate of these adenomas vary among series but can be as high as 49% [7, 8]. Some centers recommend postoperative radiation therapy routinely to all patients with NFPA, even in those with apparently complete resection of their tumors, whereas others do so on a more individual basis taking into account the size and location of the remnant and the pituitary hormone status [7–9].

Despite being the most frequent pituitary tumors, NFPAs are the least studied. The purpose of the present study was to evaluate the clinical, biochemical, imaging, and outcome characteristics of a large group of patients with NFPA who had been diagnosed and treated at a multidisciplinary clinic at our hospital.

## 2. Patients and Methods

A retrospective evaluation of patients with NFPAs followed at our center between March 2008 and August 2013 was carried out. All patients had been diagnosed and managed according to a protocol that included a complete medical history, assessment of anterior pituitary hormones, computerized visual fields evaluation, and magnetic resonance imaging (MRI) of the sellar region. Hormone measurements were performed by different commercially available immunoassays. Central hypocortisolism was defined as a morning serum cortisol <5 *μ*g/dL. The diagnosis of central hypothyroidism was based on the finding of a free T4 (FT4) <0.6 ng/dL in the presence of a low or inappropriately normal thyroid stimulating hormone (TSH). Hypogonadotropic hypogonadism was diagnosed when estradiol or testosterone levels were <20 pg/mL and 300 ng/dL, respectively, in the presence of normal or low levels of luteinizing hormone (LH) and follicle stimulating hormone (FSH). Prolactin (PRL) measurements were carried out on previously diluted sera (1 : 100) to rule out underestimation by virtue of the hook effect. Panhypopituitarism was defined as the presence of three or more pituitary hormone deficiencies.

All patients had preoperative and several postoperative MRIs. Patients usually have their first postoperative MRI 6 months after pituitary surgery. Tumor volume was calculated using the Di Chiro and Nelson formula [10].

Surgery was performed by the same three pituitary neurosurgeons (GG, BLF, and EZ) using in most cases the microscopic transsphenoidal approach; the transnasal or sublabial routes were chosen according to the size and location of the adenoma. The transcranial approach was reserved for patients with invasive and giant tumors (i.e., >4 cm). Detailed immunohistochemistry (IHC) analysis was available in 110 patients and included immunostaining for pituitary transcription factor-1, steroidogenic factor-1, estrogen receptor *α*1, LH, FSH, corticotropin (ACTH), growth hormone (GH), PRL, and TSH.

Three-dimensional, conformal, external beam radiotherapy (XRT) was administered by means of a lineal accelerator, at a mean total dose of 52 Gy (range 50–57), delivered as 2–2.5 Gy daily fractions, 5 days a week, over 5 weeks. Optic apparatus radiation dose was kept below 50.4 Gy using a multileave collimator.

### 2.1. Statistical Analysis

Quantitative variables were described as means ± standard deviation (SD) or medians and interquartile ranges (IQR) according to their distribution, which was ascertained by means of the Shapiro-Wilks test. Proportions were used for qualitative variables (expected frequency and prevalence). To establish associations between continuous variables we used Student's *t* test, Mann-Whitney's *U* test, or Wilcoxon and for qualitative variables chi square test or Fisher's, according to the expected value in the boxes. We considered a *P* value of <0.05 as significant. We used SPSS 17 and STATA 11 as statistical software.

## 3. Results

### 3.1. Clinical Presentation at Diagnosis

Four hundred and eighty-five patients with NFPAs (54% males, 46% females, with a mean age of 53.3 ± 13.6 years) were registered in our database from 2008 to 2013. The median followup at the time of registry was 6.5 years (IQR 4.4–10.6). The most common clinical findings were bitemporal hemianopia (87.2%) and headache (66%). Pituitary apoplexy, presenting not only as imaging evidence of hemorrhage, but also clinically with hemodynamic instability and/or visual abnormalities, was documented in 37 of the patients (8%); oculomotor palsies were present in 14 (2.9%) of them. In 30 patients (6.2%) the adenoma was an incidental finding (Table 1).

**Figure 1:**
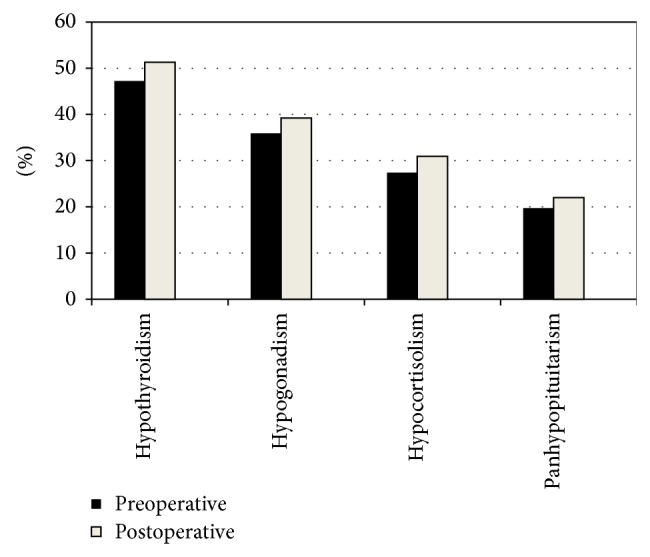
Prevalence of pituitary hormone deficiencies before and after surgery.

Regarding hormone deficiencies, 47.2% of the patients had biochemical evidence of central hypothyroidism, 35.9% had hypogonadotropic hypogonadism, and 27.4% were hypocortisolic (Figure 1). Eighty-seven patients (19.7%) had three or more hormone deficiencies, while 281 (57%) did not have any hormonal deficit (Figure 1). In 144 patients (29.7%) PRL was mildly elevated (range 25–100 ng/mL).

The median tumor volume at diagnosis was 10306 mm^3^ (IQR, 4398–15791). MRI at diagnosis showed that in 93 patients (19.1%) the adenoma was purely intrasellar, 281 (57.9%) had suprasellar or parasellar extension, and 95 (19.6%) had a giant adenoma (>4 cm).

### 3.2. Surgical Treatment and Tumor Recurrence

In 19 of the 485 patients (14.2%) included in the cohort, surgery had not taken place by the time this paper was written. These 19 patients had stable lesions, without impending irreversible visual abnormalities. In 5, aged 76 and older, surgery was deemed unnecessary and relatively contraindicated because of cardiopulmonary reasons; two of them were lost to follow-up shortly after diagnosis, whereas in the remaining 3, slight increments in tumor size occurred 4–7 years after diagnosis. In 3 women and 2 men aged 25 and younger, surgery was not performed because of fertility concerns; two were lost to followup, two showed a 20–50% increment in tumor size, and, in one, tumor size remained stable 4–6 years after diagnosis. Of 9 patients who were waiting for surgery, 6 were lost to followup after diagnosis, in 2 the adenoma remained stable, and, in one, the lesion increased in size. Thus, 10 were lost to followup, 6 showed clear evidence of tumor growth, and, in 3, the adenoma remained stable.

**Figure 2:**
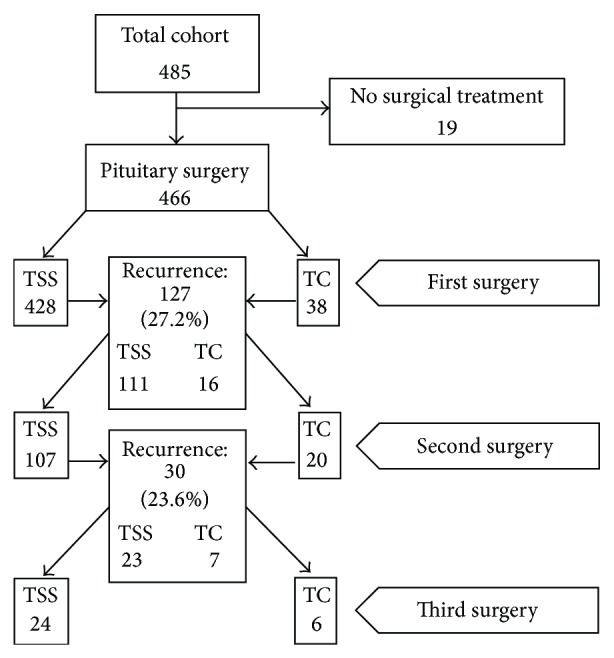
Number of patients undergoing one, two, and three surgical procedures and the corresponding persistence rates.

A total of 466 patients (96%) had undergone pituitary surgery: 428 (91.8%) via a transsphenoidal (TSS) microscopic approach and 38 (8.1%) via the transcranial (TC) route (Figure 2). A second pituitary surgery was performed in 127 patients (27.2%) found to have tumor persistence, a median of 8 years (6-13) after the initial intervention (Figure 2). Tumor persistence was documented in 30 of these 127 subjects and they underwent a third operation (24 TSS, 6 TC) (Figure 2). A fourth pituitary operation was performed in two exceptional cases. Tumor persistence occurred in 26% and 21.5% after the first and second TSS procedures, respectively, and in 42% and 35% after the first and second TC operations, respectively. Median tumor volume decreased from 10306 mm^3^ (IQR, 4398–15971) to 1413 mm^3^ (IR 419–5273) after the first surgery, which represents an 86% reduction of the initial mass, (*P* < 0.01).

As compared to patients in whom a complete tumor resection was possible, patients with adenoma persistence had larger lesions at diagnosis (14137 mm^3^ [IQR, 7916–22449] versus 9100 mm^3^ [IQR, 4188–14137] *P* = 0.01); had a higher prevalence of giant adenomas (29.6% versus 17%, *P* = 0.003); and a higher frequency of cavernous sinus invasion (68% versus 55%, *P* = 0.01). Upon a multivariate analysis including age, gender, the presence of a giant tumor, and cavernous sinus invasion, only these last two variables were significantly associated with tumor persistence (giant tumor OR 1.95, 95% CI 1.19–3.19, *P* = 0.001; cavernous sinus invasion OR 1.56, 95% CI 1–2.44, *P* = 0.04) (Table 2).

Most patients showed a significant improvement in headaches and visual field abnormalities after surgery. Pituitary hormone deficiencies on the other hand not only persisted but also increased in frequency after surgery since an extra 3.5%, 4.1%, and 3.3% developed central hypocortisolism, central hypothyroidism, and hypogonadotropic hypogonadism, respectively (Figure 1). Permanent diabetes insipidus developed in 3.3% of the patients after initial surgery. Both, multiple surgeries and radiotherapy increased the chances of developing hypopituitarism. The combination of these treatments resulted in a total of 65% of the patients with at least one hormone deficit upon last followup. Five patients died due to complications after TC surgery, one after the first operation and four after reintervention.

### 3.3. Immunohistochemistry

Immunohistochemistry (IHC) was available in 110 patients. Based on IHC for pituitary transcription factor-1, steroidogenic factor-1, estrogen receptor *α*1, LH, FSH, ACTH, GH, PRL, and TSH, 48 of these tumors (43.6%) were categorized as null-cell adenomas, 54 (49%) as gonadotroph adenomas, and 8 (7.2%) as silent adenomas (4 stained for ACTH, 2 for PRL, and 1 each for GH and TSH). The clinical, biochemical, and tumoral characteristics of these tumors are depicted in Table 3. Patients with silent adenomas were younger and manifested less frequent signs of visual field defects than null-cell adenomas or gonadotrophinomas.

### 3.4. Radiation Therapy

Fifty-one patients (45% women, mean age of 56.2 ± 11.7 years) received XRT two to 8 months after the last pituitary surgery and were followed for a median of 5 years. The outcome of these patients was compared to a group of 61 patients matched for age, gender, remnant size, and cavernous sinus invasion who were not radiated. Cavernous sinus invasion was present in 71%. Median tumor volume decreased progressively from a baseline value (after last surgery, prior to XRT) of 1601 mm^3^ (697–1538), to 1124 mm^3^ (382–2638) and 816 mm^3^ (92–1866) after 3 and 5 years of followup, respectively (*P* = 0.01); tumor volume reduction after 5 years of followup was 50%. Lineal regression analysis revealed a tumor volume reduction of 388 mm^3^ per year (95% CI: −714 to −62, *P* = 0.02). The estimated tumor progression rate was 4% in patients who received XRT and 29% in those who were not radiated (OR 0.10, 95% CI 0.01–0.04, *P* = 0.02). At 5 years followup, TSH, LH/FSH, and ACTH deficiency was present in 80%, 95%, and 62%, respectively. No cerebrovascular events, cases of optic neuritis, secondary tumors, or deaths were recorded among patients who received XRT.

### 3.5. Pharmacological Treatment

Twenty-six patients received pharmacological treatment after 3 unsuccessful surgical attempts. All of these subjects had very large or giant adenomas with significant supra- or parasellar extension. In 23 (11 women and 12 men), cabergoline (CBG) was used at doses that ranged from 1.5 to 3 mg per week. Four of these 23 patients had been previously unsuccessfully treated with Octreotide LAR 20 mg monthly for 3 to 6 months. Of the 17 patients who had been treated for longer than 6 months (range 6–18 months), two (11.7%) showed a greater than 20% reduction in tumor volume, whereas in 12 (70.5%) the adenoma remained stable and in 3 (17.6%) the tumor continued to grow. Temozolomide was tried in three subjects whose tumors were indeed very large yet did not have a clear cut imaging evidence of metastasis; tumor shrinkage was observed in one, whereas the other two have stable lesions.

## 4. Discussion

This constitutes one of the largest series of patients with NFPAs diagnosed, treated, and followed at a single center. Our results highlight the complexities in the diagnosis and management of this condition. Despite being the most frequent pituitary tumors, NFPAs have not been studied as extensively as their functional counterparts. Since they do not produce a hormonal hypersecretion syndrome, NFPAs present with signs of symptoms related to the mass effect of the lesion such as headache and visual abnormalities as well as with clinical manifestations of pituitary hormone deficiencies [1–3]. Indeed, most series published to date describe headaches and visual field defects as the two most common presenting complaints of NFPA, occurring in 40–60% and 60–80% of patients, respectively [1–3, 7]. Notable exceptions to this constitute the series of Nomikos et al. [11] and of Dekkers et al. [12]. The former is a large retrospective study assessing pituitary function in NFPAs before and after pituitary surgery in which only 9.7% and 31% of the subjects presented with headaches and visual field disturbances, respectively [11]. The latter is a study designed to evaluate the natural history of untreated NFPAs without surgical intervention that found headaches and visual field abnormalities to be present in 7% and 14%, respectively [12]. These two studies, although undoubtedly relevant because of the large number of patients they analyzed, have specific selection biases that prevent comparisons with other series. It is noteworthy that, although signs of chiasm compression were common in our series, in consonance with previous studies, the prevalence of oculomotor palsies was rather low even among patients with cavernous sinus invasion [1, 2, 7]. This is clinically important because the presence of signs of cranial nerve involvement in the setting of a pituitary mass should always prompt a search for alternative diagnosis like infiltrative diseases and metastasis to the sellar region. The low prevalence of incidentally found adenomas is due to the fact that this is a referral center experience. On the other end of the clinical spectrum, pituitary apoplexy was the presenting feature in 8% of our patients, a prevalence similar to that reported in other series [13].

Two-thirds of the patients in our cohort had tumors that extended superiorly towards the optic chiasm or laterally into the cavernous sinus; only 20% had purely intrasellar macroadenomas and another 20% of the subjects were categorized as having giant adenomas. As expected, larger and more invasive tumors had a higher recurrence/persistence rate after primary surgery than smaller noninvasive lesions. In general, this is in agreement with the literature, although the methods to assess adenoma size (volume versus diameters) and to define invasiveness among previously published reports vary widely and preclude a formal comparison [11, 12, 14, 15].

Careful immunohistochemical analysis of NFPAs reveals that a significant proportion of these tumors do in fact synthesize pituitary hormones despite the absence of a clinically apparent endocrine hypersecretion syndrome [5, 6]. IHC available in a subset of our patients revealed that almost 50% of their tumors stained for gonadotropins or their subunits, whereas a little over 40% of the tumors were categorized as null-cell adenomas. In accordance with previously published studies, we could not find any differences in the clinical presentation or course between gonadotrophinomas and null-cell adenomas. A few of the tumors available for IHC in our cohort immunostained for other pituitary hormones like GH, PRL, TSH, and ACTH. Some but not all studies have found these “silent” somatotrope, lactotrope, thyrotrope, and corticotrope adenomas behave more aggressively than the more common gonadotrophinomas or null-cell adenomas [16–19]. We found such an unusual aggressive clinical behavior only in a case of a silent GH adenoma.

Few observational studies looking at the natural course of untreated NFPAs have been published [12, 20, 21]. In general, these studies have included small numbers of patients followed for periods that range from 20 to 85 months and the proportion of them exhibiting tumor growth varies between 20 and 50%; adenoma regression is reported in 11% [7]. In our cohort 19 subjects had not been operated and had been followed for 4–7 years because of several reasons (advanced age, cardiopulmonary contraindications for surgery, and fertility preservation). Unfortunately, 10 of these subjects were lost to followup; therefore we cannot draw solid conclusions regarding the natural course of untreated NFPAs based on our data.

The treatment of choice for NFPA continues to be TSS. When performed by an experienced neurosurgeon, even large lesions with significant suprasellar extension can be successfully removed using this approach [7]. Recurrence or regrowth of the adenoma has been reported to occur in 10–40% of the patients, depending on whether or not adjunctive radiotherapy is used [12, 14, 16, 17, 22]. Adenoma regrowth is strongly influenced by the presence of a tumor remnant after initial surgery, which is a common occurrence in NFPA. In this regard, a recent report by Reddy et al. found the rate of adenoma regrowth to be only 6.9% in cases with complete tumor resection, compared to over 40% for patients in whom a remnant had been left behind, being somewhat higher for those patients with extrasellar remnants than in those with purely intrasellar lesions [23]. In the present series we describe the outcome of 466 pituitary operations, the majority of which were performed transsphenoidally. In over 70% of our patients the initial surgical procedure was apparently successful in removing the adenoma, although the presence of a small remnant in these cases cannot be ruled out completely. After the first surgical procedure, tumor volume was significantly reduced by 86%. Tumor recurrence or persistence was detected in 27%, and all of these subjects underwent a second pituitary operation, mostly via the transsphenoidal approach. Not unexpectedly, patients with larger and more invasive lesions were significantly more likely to experience tumor recurrences upon both uni- and multivariate analysis.

As has been the case in other series, visual field abnormalities and headaches improved significantly after surgery. Data regarding recovery of pituitary function after surgical treatment of NFPA is controversial. Some studies report a moderate decrease in the incidence of pituitary hormone deficiencies after surgery [11, 14, 15, 24, 25] whereas others like ours show either no postoperative change or even a further increment in the rate of hypopituitarism [12, 24–26]. This apparent discrepancy stems from variations in the design of these studies, particularly in the duration of postoperative followup.

The proven efficacy of XRT in preventing tumor regrowth has to be weighted against it's potential side effects, particularly the induction of pituitary hormone deficiencies and the risk for cerebrovascular events and, according to some authors, an increased mortality risk [9, 27–29]. In our experience XRT was indeed associated with a high rate of hypopituitarism; however, we did not observe a single case of cerebrovascular accident or optic neuritis and all radiated patients are alive to date. Although XRT should not be indicated in patients with complete adenoma removal, in patients with tumor remnants, particularly those located in surgically inaccessible locations such as the cavernous sinus, it is a safe and effective treatment alternative.

Both mRNA expression and immunohistochemical studies have shown that NFPAs express somatostatin (sstr 3 and sstr 5) and dopamine receptors (D2R) in variable proportions and subtypes [29–32]. Evaluating the efficacy of the pharmacological treatment of NFPA is hampered by the lack of a measurable tumor marker as what occurs in functioning tumors [33]. The ideal experimental design should be that of a prospective study comparing the pharmacological intervention with placebo over a long period of time (several years), using serial MRIs, since these tumors grow rather slowly. Patients included in such a study should ideally have tumors expressing either somatostatin or dopamine receptors. To our knowledge, no such study has either been published or been conducted at the moment. Available data suggest that treatment with somatostatin analogs reduces tumor volume in barely 12% of the patients [34]. The clinical experience with CBG has yielded somewhat better results with more than 25% reduction in tumor volume in 30–50% of the patients treated for 6–12 months, particularly in those with tumors expressing D2R [29, 35, 36]. Our experience with Octreotide LAR is so limited that it does not allow us to draw any conclusions. We treated with CBG some patients who despite several surgical interventions persisted with significant tumor remnants and although in the majority of them the lesion remained stable, only a few showed an objective reduction in adenoma size.

## 5. Conclusion

Although TSS remains the treatment of choice for NFPAs, complete tumor removal is frequently not possible and recurrence rates are considerable even in specialized centers. A multidisciplinary approach is mandatory in order to address specific issues such as postoperative hypopituitarism and the need to appropriately indicate adjunctive therapies like XRT or pharmacological treatment. Our results underscore the importance of long-term surveillance of these patients in order to identify and treat recurring lesions.

## Figures and Tables

**Table 1:** Baseline characteristics of the patients with NFPA (*n* = 485).

Age, mean ± SD	53.3 ± 13.6
Female, *n* (%)	223 (46)
Tumor volume at diagnosis, mm^3^ (IQR)	10306 (4398–15791)
Headache, *n* (%)	323 (66)
Giant tumor (>4 cm), *n* (%)	95 (19.6)
Visual deficit, *n* (%)	423 (87.2)
Supra- and parasellar extension, *n* (%)	281 (57.9)
Apoplexy, *n* (%)	37 (8)
Cranial nerve palsy, *n* (%)	14 (2.9)
Incidentaloma, *n* (%)	30 (6.2)

**Table 2:** Multivariate analysis of features potentially associated with tumor recurrence.

Variable	OR	95% confidence interval	*P*
Age	0.99	0.97–1	0.25
Gender	1.29	0.84–1.97	0.23
Giant adenoma	1.95	1.19–3.19	0.001
Cavernous sinus invasion	1.56	1–2.44	0.04

**Table 3:** Comparison of clinical, endocrine, and tumoral features among patients with null-cell, gonadotrophin-producing, and silent adenomas.

	Null-cell (*n* = 48)	Gonadotrophinomas (*n* = 54)	Silent adenomas (*n* = 8)	*P*
Age (years)	53.2 ± 14.9	58.3 ± 12.2	41.4 ± 14	0.05
Females	24 (50%)	33%	62%	0.11
Visual field defects	85%	90%	50%	0.01
Hypothyroidism	22	25	3	0.9
Hypogonadism	18	19	1	0.38
Hypocortisolism	12	20	1	0.35
Panhypopituitarism	8	12	0	0.36
Tumor volume	13819 mm^3^	11162 mm^3^	4673 mm^3^	0.27
Median (IQR)	(8202–20168)	(6900–19476)	(4188–15079)	
Supra- or parasellar extension	56%	68%	50%	0.8
Giant	27%	24%	0%	0.24
Recurrent	15	18	1	0.56
Apoplexy	3	1	1	0.34
